# Middle aged male with pulmonary tuberculosis and refractory hypercalcemia at a tertiary care centre in South East Asia: a case report

**DOI:** 10.4076/1757-1626-2-6316

**Published:** 2009-07-06

**Authors:** Azra Rizwan, Najmul Islam

**Affiliations:** Department of Medicine, Aga Khan University & HospitalStadium Road, Karachi 74800, P.O. Box 3500Pakistan

## Abstract

55-year male of Asian descent presented with weight loss, lethargy, drowsiness and low grade fever without cough. Examination revealed crackles in the chest but no focal neurological deficit. Chest X ray revealed an infiltrate consistent with tuberculosis. Biopsy of infiltrate was negative for malignancy. Corrected calcium level revealed parathyroid independent hypercalcemia. Further diagnostic work up for drowsiness and hypercalcemia was normal. Despite receiving hydration and pharmacotherapy for his hypercalcemia, his condition failed to improve. When steroids were started, the patient’s calcium levels and symptomatology resolved. Tuberculosis causing hypercalcemia is uncommon. Steroids are useful agents, particularly in refractory cases.

## Introduction

Hypercalcemia has long been known to be associated with tuberculosis, with a recent increase in its occurrence possibly related to widespread accessibility to diagnostic testing [1,2]. Generally speaking, the hypercalcemia is mild and asymptomatic [2,3]. We aim to describe a case of a middle-aged gentleman with refractory hypercalcemia associated with tuberculosis of the lungs.

## Case presentation

This is a description of a 55-year-old male, banker by profession and of Asian descent, an Urdu speaking Pakistani. He was a long-standing smoker, having smoked 3 packs per day for 30 years prior to presentation. He denied any form of alcohol intake and had been found to have type 2 diabetes 17 years prior to presentation. There was no known history of tuberculosis or any form of calcium disorder in parents, siblings or children. He weighed 47 kg and was 1.65 mt tall.

He presented in December 2007 with a 6-month history of unplanned weight loss, of up to 13 kg, associated with recent onset weakness and drowsiness, low grade fever but no cough. Clinical examination revealed a normotensive, lean individual, with crackles in his left upper chest but no focal neurological deficit.

An X- ray of his chest revealed a left lingular infiltrate. Brochoalveolar lavage produced a smear positive and culture positive tuberculosis that was pan sensitive. Biopsy of the infiltrate was negative for malignancy.

His serum calcium level was high, (Table 1), with the initial corrected calcium level at 13.2 mg/dl and a low parathormone level, with normal 25-OH Vitamin D levels and a high 24 hour urine calcium. His serum phosphate was normal. A diagnostic work up for drowsiness and hypercalcemia, (Table 2), revealed a normal serum sodium. Serum magnesium was initially low and was corrected to a normal level. Thyroid function tests were initially consistent with hyperthyroidism, with the thyroid scan showing negligible uptake. They subsequently normalized within 4 weeks. Serum protein electrophoresis, liver function tests, angiotensin converting enzyme (ACE) levels and a morning cortisol level were normal.

**Table 1.** Initial Investigations

Chest x ray	Left Lingular Infiltrate	
Broncho-alveolar lavage	Smear Positive and Culture Positive Tuberculosis- Pansensitive	
Biopsy of infiltrate	Negative for Malignancy	
	**Patient Result**	**Normal Range**
Initial corrected calcium level	13.2	8.6 - 10.5
Parathormone level	7.76	16 - 87
25 OH vitamin D	34.5	> 30
24 hour urine calcium	302	100 - 300

**Table 2.** Diagnostic work up for drowsiness and hypercalcemia

	Patient Result	Normal Range
Serum sodium	137	136 - 146
Serum magnesium	1.4	1.9 - 2.5
Serum magnesium after correction	2.1	1.9 - 2.5
Thyroid function tests		
TSH	1.18	0.27 - 4.2
FT4	1.65	0.93 - 1.7

	Patient Result	Normal Range
**SerumProtein Electrophoresis**		
Total Protein	5.80 g/dl	5.7 - 7.75
Albumin	2.93 g/dl	3.5 - 4.0
Gamma	1.17 g/dl	0.71 - 1.45
**Liver Function Tests**		
SGPT	14	0 - 55
Alkaline Phosphatase	82	28 - 124
ACE Level	10	8.0 - 52.0
Serum AM Cortisol	34.90 ug/dl	4.2 - 38.4
Serum Phosphate	3.1 mg/dl	2.7 - 4.8
CSF D/R	200 mg/dl	15 - 45
**Vasculitic Work Up**		
ANA	Negative	
ASMA	Negative	
AMA	Negative	
MRI Brain	No Tuberculomas	

MRI brain was negative for tuberculomas. CSF D/R revealed a high protein content, while routine and AFB cultures of the CSF were negative. Vasculitic work up was negative.

Hence, a diagnosis of hypercalcemia secondary to pulmonary tuberculosis was made.

The patient was hydrated aggressively, followed by diuresis with intravenous furosemide, 40 mg, once the patient had been well hydrated. Intravenous pamidronate 90 mg was administered twice over a period of two months, as well as daily calcitonin injections at 240 IU subcutaneously twice daily. A nephrology consult revealed that dialysis was not feasible because of non availability of a calcium free dialysate. Subsequently, steroids were started at 40 mg/day, while his anti tuberculous medication was continued, including isoniazid 300 mg, rifampicin 600 mg, ethambutol 1200 mg, pyrazinamide 1500 mg per day.

**Figure 1.**
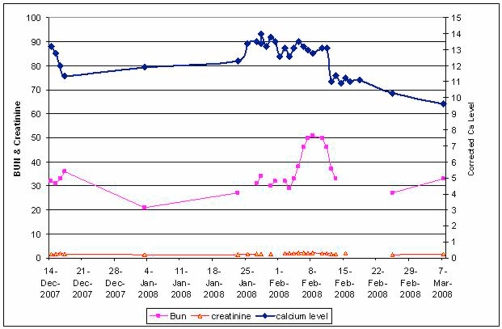
Graph showing the serial changes in blood levels of calcium, creatinine and bun.

Serum calcium fluctuated between 12.0 - 14.2 for a period of two and a half months and his drowsiness, disorientation & hypercalcemia failed to resolve with the initial measures. Once steroids were started, the calcium levels gradually began to resolve (Figure 1), as did the patient’s confusional status.

## Discussion

Primary hyperparathyroidism and malignancy remain the most common etiologies of hypercalcemia, accounting for 90% of all cases [2]. Chronic granulomatous disease is well known to cause hypercalcemia [1,4]. Tuberculosis and fungal infections are subtypes of chronic granulomatous disease that are more prevalent in the developing countries [1,4], or the immunocompromised individuals across the globe [5-8]. Sarcoidosis is a form of chronic granulomatous disease seen more commonly in Caucasians [9]. These entities need to be considered in the differentials, in the appropriate context. Chronic granulomatous disease is associated with a parathyroid independent hypercalcemia, resulting from the 1,25 dihydroxyvitamin D producing granulomas. The non availability of the 1,25 dihydroxyvitamin D level precluded us from ordering the test, a limitation of the report because it is high levels of this biochemical marker that would have helped to confirm the etiology.

Our patient did exhibit a transient thyroiditis, as evidenced by the negligible uptake on thyroid scan. Thyroiditis has been documented in the literature to be associated with tuberculosis [10] , with the hypercalcemia resulting from the hyperthyroidism. However, the calcium levels had remained elevated despite resolution of the thyroiditis. Other causes such as adrenal insufficiency, multiple myeloma and sarcoidosis that may have additionally contributed were essentially ruled out through a normal morning cortisol, serum protein electrophoresis and ACE levels, respectively. There had been no history of recent ingestion of antacids, vitamin A, D or calcium supplements, nor had there been any reported use of thiazide diuretics or lithium.

Tuberculosis causing hypercalcemia is an uncommon but well recognized phenomenon, particularly in the Asian subcontinent where concomitant vitamin D deficiency makes the occurrence even less likely [11-13]. This degree of symptomatic hypercalcemia is even rarer [14], which in this patient manifested as prolonged alteration in mentation. In most cases, the hypercalcemia responds to simple conventional measures of hydration and diuresis [15]. Dialysis is recommended in resistant cases but could not be availed due to the current non availability in our unit of a calcium free dialysate. Steroids are useful agents to use, particularly in refractory cases, where they act through their anti inflammatory effects on the vitamin D producing granulomas & through inhibition of the one alfa hydroxylase enzyme that converts 25 OH vitamin D to 1,25 Dihydroxyvitamin D [15].

## Conclusion

Knowledge of the association between tuberculosis and hypercalcemia, plus its mechanism of action is important in order to correct the symtomatology resulting from elevated calcium levels. Steroid therapy needs to be instituted early in cases of hypercalcemia complicating tuberculosis.

## Abbreviations

ACE: Angiotensin Converting Enzyme
AFB: Acid Fast Bacilli
CSF D/R: Cerebrospinal Fluid Detailed Report
MRI: Magnetic Resonance Imaging
25-OH vitamin D: 25 hydroxyvitamin D
